# Subgenome-Resolved Analysis and Regulatory Divergence of UDP-Glycosyltransferases in Allotetraploid *Panax ginseng*

**DOI:** 10.3390/genes17091140

**Published:** 2026-09-17

**Authors:** Qizhan Guo, Xin He, Lingping Yang, Xiaojuan Tian, Mingxu Wu, Ting Zhang, Liying Feng, Anqiang Jia

**Affiliations:** 1 Institute of Herbgenomics, Chengdu University of Traditional Chinese Medicine, Chengdu 611137, China; 20261006@cdutcm.edu.cn (Q.G.);; 2 Innovative Institute of Chinese Medicine and Pharmacy, Chengdu University of Traditional Chinese Medicine, Chengdu 611137, China; 3 School of Basic Medical Sciences, Chengdu University of Traditional Chinese Medicine, Chengdu 611137, China; 4 Yazhouwan National Laboratory, Sanya 572024, China

**Keywords:** *Panax ginseng*, UDP-glycosyltransferase (UGT), allotetraploid, subgenome evolution, regulatory divergence, stress response

## Abstract

**Background**: Polyploidization generates extensive gene redundancy, but how duplicated metabolic genes are retained and subsequently diversified remains poorly understood. UDP-glycosyltransferases (UGTs) provide a suitable system for examining this process because they participate in specialized metabolism, plant development, and environmental responses. This study aimed to characterize the retention, expansion, and regulatory divergence of the UGT family in allotetraploid *Panax ginseng* at subgenome resolution. **Methods**: We integrated telomere-to-telomere (T2T) genome annotation, phylogenetic and chromosomal analyses, duplication classification, collinearity and *Ka*/*Ks* analyses, promoter cis-acting element prediction, developmental co-expression networks, and transcriptomic responses to biotic and abiotic treatments. **Results**: A total of 212 *PgUGT* genes were identified, including 104 and 108 members in the A and B subgenomes, respectively. The family exhibited an overall near-mirrored retention pattern between the two subgenomes, accompanied by local copy-number asymmetry. Whole-genome and segmental duplication accounted for 64.2% of the family, and 99.5% of the gene pairs with valid *Ka*/*Ks* estimates had values below 1, suggesting pervasive purifying selection. *PgUGT*-containing co-expression modules were associated with bud, stem, leaf, and fruit developmental conditions, while promoter cis-acting element compositions exhibited member-specific variation. Transcriptional responses to fungal pathogens and abiotic, hormone, and chemical treatments were concentrated in particular members and local gene arrays rather than being coordinated across entire clades or subgenomes. **Conclusions**: The *PgUGT* family is characterized by extensive ancestral copy retention accompanied by local copy-number changes and copy-specific regulatory divergence. These findings provide a subgenome-resolved framework for understanding UGT family evolution in allotetraploid ginseng and prioritize candidate *PgUGT* genes for subsequent functional validation.

## 1. Introduction

Over the course of evolution, plants have developed a rich repertoire of specialized metabolites, including terpenoids, alkaloids, phenylpropanoids, and flavonoids [1,2]. These compounds contribute broadly to plant growth and development, responses to biotic and abiotic stresses, and interspecific interactions. Glycosylation is a major modification that expands the structural and functional diversity of specialized metabolites and is catalyzed predominantly by UDP-dependent glycosyltransferases (UGTs). UGTs use UDP-activated sugars as donors and transfer sugar moieties to hydroxyl, carboxyl, amino, or other acceptor groups on plant hormones, signaling molecules, and specialized metabolites, thereby altering their water solubility, stability, subcellular compartmentation, and biological activity [3,4,5,6,7]. Plant family 1 UGTs generally adopt a conserved GT-B fold. Their C-terminal region contains the plant secondary product glycosyltransferase (PSPG) box, which primarily participates in UDP-sugar donor recognition, whereas the more variable N-terminal region largely determines acceptor specificity. This structural organization enables UGTs to accommodate diverse acceptor substrates while retaining a conserved catalytic framework [8,9,10]. Whole-genome duplication (WGD), segmental duplication (large chromosomal blocks), and tandem duplication (adjacent local copies) have further driven the expansion of plant UGT families and provided a genetic basis for divergence in expression regulation and function among duplicated copies, including both homoeologs (cross-subgenome counterparts) and paralogs (within-genome duplicates) [10,11].

Ginseng (*Panax ginseng*) is a perennial allotetraploid (2n = 4x = 48) medicinal plant whose medicinal value largely relies on ginsenoside accumulation [12,13,14]. As a perennial species, it must coordinate organ development with age- and season-related physiological changes while coping with biotic stress [15,16,17]. Therefore, systematic characterization of the UGT family is crucial for understanding its evolution and functional diversification, as UGTs link small-molecule modification to growth, development, and environmental responses. With the increasing availability of high-quality reference genomes, UGT gene families have been systematically characterized in cotton (*Gossypium hirsutum*) [18], Japanese honeysuckle (*Lonicera japonica*) [19], and *Epimedium* species [20]. These studies have provided a comparative framework for plant UGT families. However, how allotetraploidization and subsequent duplication/fractionation shaped *PgUGT* retention, local expansion/contraction, and regulatory divergence across the A and B subgenomes, particularly at T2T resolution, remains unclear. We hypothesize that extensive retention of ancestral *PgUGT* copies was accompanied by local copy-number changes and copy-specific regulatory divergence, generating candidates for functional validation.

Functional studies have shown that UGTs contribute to plant growth and development by modulating the homeostasis of phytohormones and related metabolites. In *Arabidopsis thaliana*, UGT76C1 and UGT76C2 catalyze cytokinin N-glycosylation [21]. Loss of UGT76C2 reduces cytokinin N-glycoside levels, increases the sensitivity of root growth to cytokinins, and results in smaller seeds [22]. UGT73C5 glucosylates brassinolide and castasterone at the 23-O position; its overexpression lowers endogenous brassinosteroid levels and causes typical brassinosteroid-deficient phenotypes [23]. In rice (*Oryza sativa*), GSA1 (UGT83A1) contributes to grain development by modulating flavonoid metabolism, auxin levels, and cell proliferation and expansion, and its overexpression produces larger grains [24]. A systematic analysis of auxin inactivation pathways in *A. thaliana* further demonstrated that UGT-mediated glycosylation of indole-3-acetic acid (IAA) is an important component of IAA homeostasis and plant development [25]. UGTs thus act as key developmental regulators, modulating root, shoot, and seed growth via auxin, cytokinin, and brassinosteroid homeostasis.

UGTs contribute to plant responses to biotic stress by maintaining host defense-signal homeostasis, detoxifying pathogen-derived toxic metabolites, or facilitating the accumulation of defense-related metabolites in less phytotoxic forms. In *A. thaliana*, overexpression of AtSGT1 (UGT74F2), which encodes salicylic acid glucosyltransferase 1, increases plant susceptibility to *Pseudomonas syringae* [26]. The pathogen-responsive genes UGT73B3 and UGT73B5 participate in the AvrRpm1-triggered resistance response, and loss of either gene results in reduced resistance [27]. In cereal crops, the barley enzyme HvUGT13248 converts the *Fusarium* mycotoxin deoxynivalenol (DON) into the less toxic deoxynivalenol-3-O-glucoside (D3G); expression of HvUGT13248 in transgenic wheat enhances type II resistance to *Fusarium* head blight (FHB) by restricting disease spread within the spike [28]. In mango (*Mangifera indica*), MiUGT92A14 and MiUGT95B15 glycosylate phenolic acids and flavonoids, respectively, and the resulting glycosides show antifungal activity comparable to or greater than their aglycones, indicating that glycosylation facilitates the accumulation of defensive metabolites with reduced phytotoxicity while preserving activity [29]. Thus, the role of UGTs in disease resistance is not uniform but context-dependent, varying with the nature of the substrate and the biological function of the corresponding glycoside product.

UGTs have contrasting roles in abiotic stress tolerance. In ginseng, PgUGT84K2 catalyzes the glycosylation of indole-3-butyric acid (IBA), and its heterologous overexpression in *A. thaliana* enhances tolerance to abiotic stress [30]. In rice (*O. sativa*), cadmium (Cd) exposure induces Os79 expression, whereas loss of Os79 enhances Cd tolerance and reduces Cd accumulation, indicating that Os79 negatively regulates Cd tolerance but promotes its accumulation [31]. In *A. thaliana*, UGT79B2 and UGT79B3 are induced by cold, salt, and drought stress, and at low temperature their transcription is regulated by CBF1. Overexpression of either gene increases anthocyanin and enhances tolerance to these stresses, but this effect is lost in the anthocyanin-deficient tt18 mutant [32]. Hormone glycosylation effects also depend on substrate. UGT71C5 glucosylates ABA to ABA-GE; mutation or downregulation of UGT71C5 raises ABA levels and enhances drought tolerance, whereas overexpression reduces both [33]. UGT74E2 preferentially glycosylates IBA, and its ectopic expression alters auxin homeostasis and shoot architecture while significantly improving plant survival under drought and salt stress [34]. Collectively, increased UGT expression does not necessarily enhance stress tolerance; the effects depend on the modified substrate, the bioactivity of the glycosylated product, and the physiological context of glycosylation.

The telomere-to-telomere (T2T) reference genome of *P. ginseng* resolves into A and B subgenomes [13], thereby enabling gene-family analyses at subgenome resolution. However, the patterns of UGT subgenomic retention and local expansion, and whether conserved copies show regulatory divergence across organs and stresses, remain unclear. Here, we systematically identified *PgUGT* family members and characterized their retention, expansion, and coding-sequence evolution via chromosomal mapping, phylogenetic and syntenic analyses, and *Ka*/*Ks* estimation. We further integrated promoter cis-regulatory element analysis, transcriptomic profiles across growth and development, co-expression networks, and expression responses to biotic and abiotic stresses to characterize tissue- and condition-associated regulatory divergence among different *PgUGT* copies. Through these analyses, this study aimed to elucidate the impact of allotetraploidization on the evolution and expression regulation of the ginseng UGT family and to prioritize candidate *PgUGT*s for subsequent functional validation.

## 2. Materials and Methods

### 2.1. Genome Resources and PgUGT Identification

The T2T genome data of allotetraploid *P. ginseng* were downloaded from figshare (https://doi.org/10.6084/m9.figshare.25477741) [13]. This genome comprises two subgenomes, A and B, with a total of 24 chromosomes and 77,266 annotated protein-coding genes. The longest transcript of each gene was used for UGT identification. Using the 122 *A. thaliana* family-1 UGT protein sequences (TAIR10) as the seed set, candidate transcripts were retrieved by BLASTp v2.15.0 (E-value < 1 × 10^−5^) [35,36], and conserved domains were then confirmed with HMMER v3.4 [37] against the Pfam PF00201 domain (E-value < 1 × 10^−5^, with domain alignment length ≥ 100 aa) [38] and NCBI Batch CD-Search [39]. Physicochemical properties of the PgUGT proteins, including molecular weight, theoretical isoelectric point, and GRAVY, were calculated with ExPASy ProtParam [40].

### 2.2. Phylogenetic, Gene-Structure, and Conserved-Motif Analysis

Phylogenetic analysis used the 212 full-length PgUGT protein sequences as input. Sequences were aligned with MUSCLE v3.8.1551 [41] under the auto strategy and trimmed with trimAl (-automated1), retaining 329 informative positions [42]. A maximum-likelihood phylogeny was inferred using IQ-TREE v2.2.3 with ModelFinder selecting LG + F + R6 as the best-fit model, supported by 1000 ultrafast bootstrap replicates (-bb 1000 -bnni) [43]. The phylogenetic tree was visualized using iTOL v7, with bootstrap support circles scaled to display values between 50% and 100% [44]. Gene structures were characterized using the exon–intron annotations in the reference GFF3 file. Conserved motifs were identified with MEME Suite v5.0.5 (maximum number of motifs, 15; motif width, 6–50 aa) [45]. Integrated visualization was performed with TBtools v2.535 [46], featuring a split genomic scale (0–6000 bp expanded, >6000 bp compressed) to display gene architectures.

### 2.3. Chromosomal Localization, Duplication Type, and Collinearity Analysis

Chromosomal localization was obtained from the genome GFF3 annotation file. Protein similarity searches were performed with DIAMOND v2.1.24 at an E-value threshold of 1 × 10^−10^ [47]. Collinear blocks and duplication types were identified with MCScanX, using parameters: match_score = 50, gap_penalty = −1, match_size = 5, overlap_window = 5, max_gaps = 25, and E-value < 1 × 10^−5^ [48]. MCScanX classified *PgUGT* members into four categories: WGD/segmental, tandem, proximal, and dispersed duplications. *PgUGT*–*PgUGT* collinear pairs were further divided into three classes (A–A, B–B, and A–B) according to their subgenome relationships, and pairs assigned to the A–B class were additionally separated according to whether the two partners reside on correspondingly numbered homologous chromosome groups. The Circos program in TBtools v2.535 was used to display the collinear relationships among chromosomal genes [46,49].

### 2.4. Selection-Pressure Analysis

Selection-pressure analysis was performed on homologous *PgUGT* gene pairs within the *P. ginseng* genome. For each pair, coding sequences and their corresponding protein sequences were used as input. The Nei–Gojobori method was implemented in the TBtools v2.535 Simple *Ka*/*Ks* Calculator (NG) module [46] to compute synonymous (*Ks*) and nonsynonymous (*Ka*) substitution rates and their ratios (*Ka*/*Ks*), based on protein sequence alignment and subsequent codon alignment. Pairs with *Ka* and *Ks* equal to zero were excluded from subsequent analysis.

### 2.5. Transcriptome Data Sources, Expression Quantification, and Differential Expression Analysis

A total of 157 RNA-seq samples were included in this study, of which 145 were obtained from the NCBI SRA database [50], and 12 were generated in this study. The dataset comprised 69 growth and development samples (23 groups) and 88 stress/treatment samples (28 stress/treatment groups) covering various conditions, including pathogen infection, water treatments, nitrogen deficiency, phytohormone treatments, and exogenous ginsenoside Ro application (Table S4).

Transcript quantification was performed using the following pipeline: (1) adapter sequences and low-quality bases were removed with fastp v0.21.0 [51] with the parameter --length_required = 20 and otherwise default settings; (2) clean reads were aligned to the *P. ginseng* T2T reference genome using HISAT2 v2.2.1 [52] with the default parameters and -p 8 threads; (3) transcript abundance was quantified as TPM (transcripts per million) values using StringTie v2.1.5 [53] in reference-guided mode with the provided GTF annotation. Differential expression was analyzed with DESeq2 [54] on treatment–control pairs within each project, with significance threshold set at padj < 0.05 and |log_2_FC| ≥ 1.0.

### 2.6. Weighted Gene Co-Expression Network and Functional Enrichment Analyses

Co-expression analysis of growth and development data was performed using the WGCNA R package [55], based on a curated developmental atlas of 59 samples across 20 conditions. A signed topological overlap matrix (Signed TOM) was constructed with soft-thresholding power selected at scale-free topology *R*^2^ > 0.80. Network modules were detected using minModuleSize = 30 and mergeCutHeight = 0.25. Module–trait correlations were evaluated with Pearson’s coefficients and Benjamini–Hochberg-adjusted *p* values. Representative modules containing *PgUGT* members were then extracted. Co-expression relationships within modules were visualized using Cytoscape v3.10.4 [56].

For functional annotation, whole-genome protein sequences from *P. ginseng* were first subjected to homology-based annotation using eggNOG-mapper v2, and Gene Ontology (GO) annotations were extracted [57]. GO enrichment analysis was subsequently performed on genes in the representative co-expression modules using the clusterProfiler R package (v4.0) [58] against the background gene universe of all 35,182 protein-coding genes in *P. ginseng* with valid GO annotations, with significance declared at Benjamini–Hochberg FDR padj < 0.05.

### 2.7. Promoter Cis-Acting Element Analysis

According to the orientation of the strand on which each gene is located, the 2-kb genomic sequence upstream of the 5′ untranslated region (UTR) of all 212 *PgUGT*s was extracted from the ginseng T2T reference genome as the candidate promoter region. Cis-acting elements in the promoters were predicted using the PlantCARE database [59]. The curated 5371 element records were merged into 20 functional categories, mainly covering light-responsive, hormone-responsive, biotic and abiotic stress-responsive, and growth, development, and tissue-related elements (Table S5). The classification results for the cis-acting elements were organized and visualized using TBtools v2.535 [46].

### 2.8. Plant Materials and Transcriptome Sequencing

Four-year-old *P. ginseng* plants cultivated in Changbai Mountain, Ji’an, Jilin Province, were sampled on 2 September 2025. Fine roots (FR), primary roots (PR), leaves, and stems were collected separately. For each tissue type, samples from three individual plants with uniform growth were pooled to constitute a single biological replicate, and three independent biological replicates were prepared per tissue (totaling nine plants per tissue). All samples were immediately frozen in liquid nitrogen upon collection and stored at −80 °C until RNA extraction. Total RNA was isolated using TRIzol^®^ reagent (Invitrogen, Carlsbad, CA, USA). RNA integrity and purity were assessed using an Agilent 2100 Bioanalyzer (RIN ≥ 7.5) and NanoDrop spectrophotometer (OD260/280 ≥ 1.8), respectively. Paired-end sequencing (150 bp read length) was performed on the MGISEQ-2000 platform (BGI Genomics, Shenzhen, China) by Benagen Technology Co., Ltd. (Wuhan, China), generating at least 6 Gb of clean data per sample.

## 3. Results

### 3.1. The PgUGT Family Shows Balanced Retention Between A and B Subgenomes After Allotetraploidization

**Figure 1:**
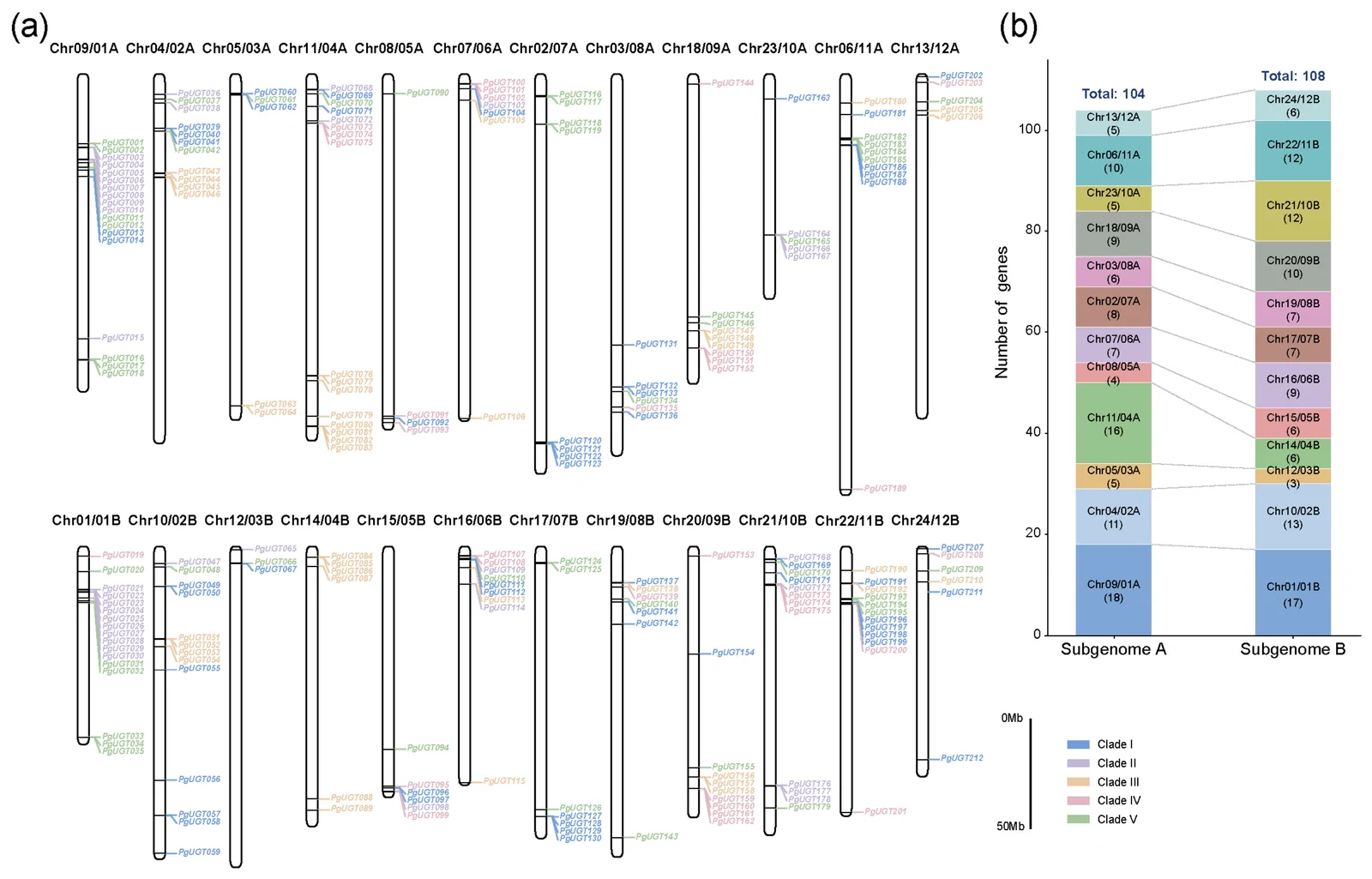
Chromosomal distribution of the *PgUGT* family. (**a**) Physical localization of the 212 *PgUGT* genes on the 24 chromosomes of allotetraploid ginseng; chromosomes are arranged in A/B subgenome pairs, and gene names are colored by clade (Clade I–V). (**b**) Correspondence of *PgUGT* numbers on homologous chromosomes between the A and B subgenomes.

A total of 212 *PgUGT* genes were identified in *P. ginseng* (Table S1). These genes were unevenly distributed across the 24 chromosomes (Figure 1a), with most members clustering toward the distal ends and fewer in pericentromeric regions. Among them, Chr09/01A harbored the most members (18), followed by Chr01/01B (17); together, these two chromosomes accounted for 16.5% (35/212) of the family. In contrast, Chr12/03B and Chr08/05A contained the fewest members, with only three and four, respectively.

At the subgenome level, the *PgUGT* family was distributed relatively evenly, with 104 members (49.1%) in subgenome A and 108 (50.9%) in subgenome B. The family also showed clear copy-number asymmetry between some homologous chromosome pairs. For example, Chr11/04A and Chr14/04B contained 16 and six *PgUGT*s, respectively, whereas Chr23/10A and Chr21/10B contained five and 12, respectively. Because the direction of copy-number asymmetry varied among homologous chromosome pairs, these patterns do not indicate a simple genome-wide subgenome bias but instead reflect local differences in *PgUGT* retention and expansion.

The 212 PgUGT proteins ranged from 109 to 1120 aa in length, with a mean molecular weight of 48.78 kDa, a mean theoretical isoelectric point (pI) of 5.94, and a mean grand average of hydropathicity (GRAVY) of −0.134 (Table S1). Proteins encoded by subgenomes A and B had median molecular weights of 51.26 and 51.85 kDa and median theoretical pI values of 5.82 and 5.83, respectively. Neither molecular weight nor pI differed significantly between the two subgenomes (Mann–Whitney U test, *p* = 0.249 and *p* = 0.918, respectively). Thus, despite chromosomal and local copy-number variation, the basic physicochemical properties of proteins from the two subgenomes were broadly similar.

### 3.2. Phylogenetic Relationships and Mirrored Retention of PgUGTs

**Figure 2:**
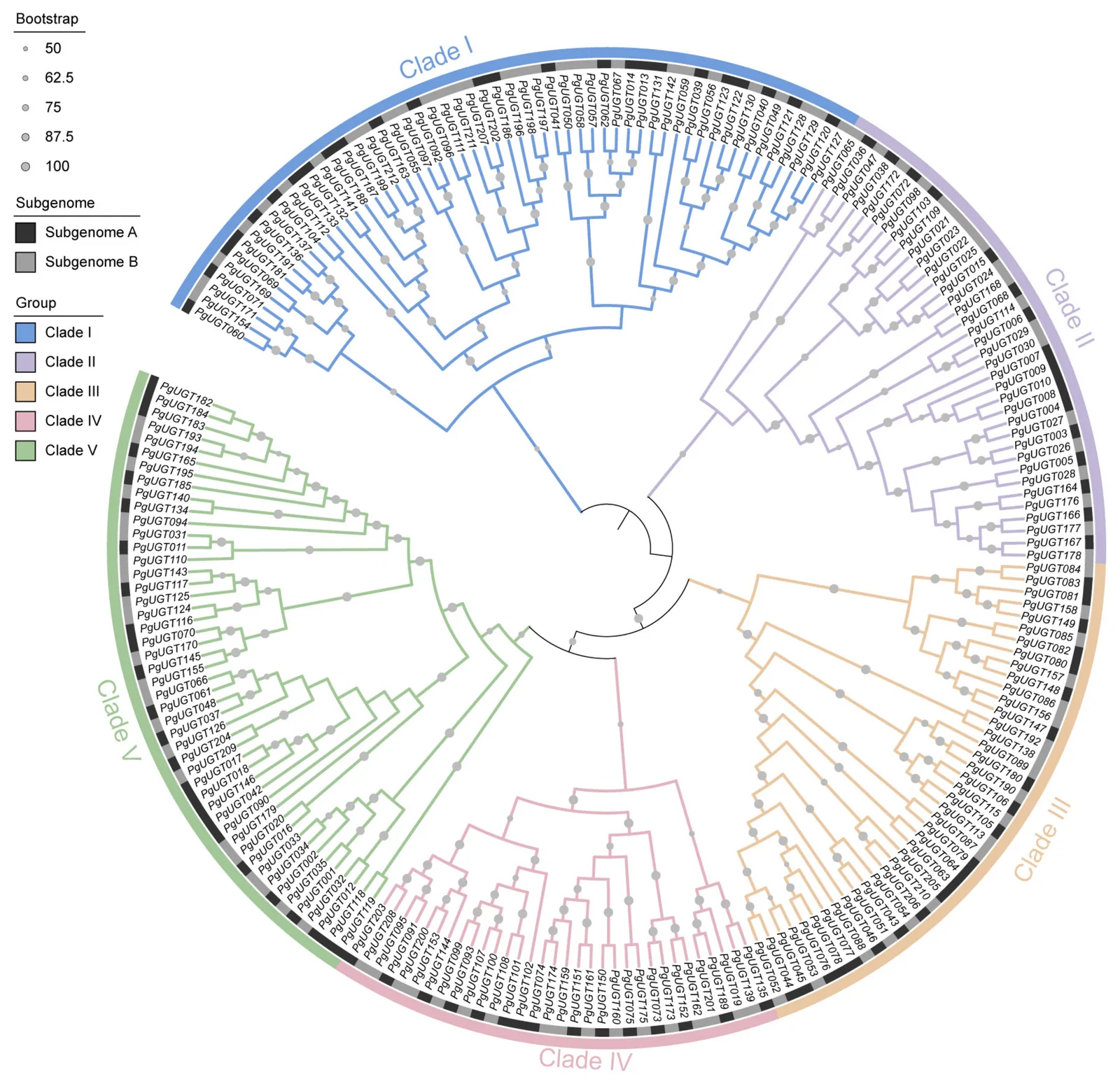
Maximum-likelihood phylogenetic tree of the 212 PgUGT proteins. Colors denote the five major clades (I–V). The outer ring shows subgenome A (black) and B (gray) assignment. Node size reflects ultrafast bootstrap support (50–100%; values <50% are not displayed). The same black/gray color scheme is used throughout.

A maximum-likelihood tree based on the full-length protein sequences divided the 212 *PgUGT*s into five major clades (Figure 2) [41,42,43]. The five clades contained 32 (Clade IV), 37 (Clade II), 41 (Clade III), 47 (Clade V), and 55 (Clade I) members, respectively (Table S1). All five clades contained members from both subgenomes, with A/B ratios generally close to 1:1, demonstrating balanced reciprocal retention of homoeologous copies across subgenomes following allopolyploidization.

Integration of phylogenetic relationships with chromosomal distribution revealed a near-mirrored retention pattern between the A and B subgenomes. Among the 39 cross-subgenome collinear pairs, 28 were recovered as reciprocal sister clades in the phylogeny with 100% ultrafast bootstrap support (Figure 2), located in corresponding homologous chromosomal regions. Representative pairs verified against the final collinearity table included *PgUGT040*/*PgUGT049*, *PgUGT181*/*PgUGT191*, *PgUGT180*/*PgUGT190*, and *PgUGT203*/*PgUGT208*. On the homologous chromosome pair Chr06/11A and Chr22/11B, the clade composition and linear arrangement of *PgUGT* members were also broadly similar. However, this mirrored pattern was not uniform across all homologous chromosomes. Chr11/04A and Chr14/04B contained 16 and six *PgUGT*s, respectively, whereas Chr03/08A and Chr19/08B also differed in copy number and gene position. These local asymmetries are consistent with differential retention, local segmental duplication, or chromosomal rearrangement after polyploidization. Together, the correspondence between phylogenetic topology and chromosomal position supports widespread retention of ancestral *PgUGT* copies after allopolyploidization, accompanied by local differences in retention and copy number [13,60]. These results indicate that most ancestral *PgUGT* copies were retained after polyploidization, whereas local copy-number asymmetry reflects subsequent lineage-specific expansion.

### 3.3. Conserved Motifs and Gene-Structure Features of PgUGTs

**Figure 3:**
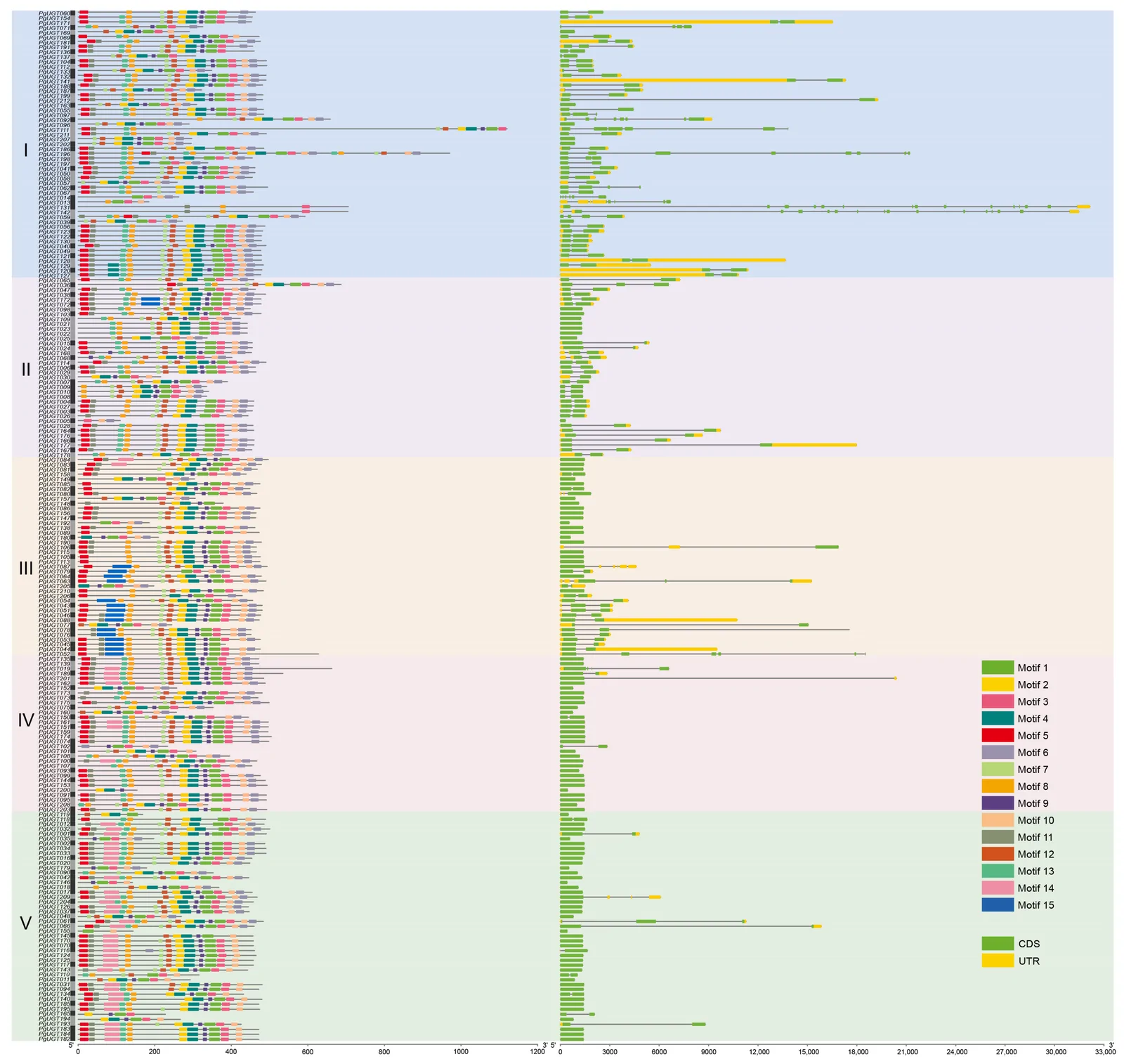
Integrated phylogeny, conserved motifs, and gene structures of the *PgUGT* family. (**Left**) Phylogenetic tree of the 212 *PgUGT* genes. (**Middle**) Distribution of the 15 conserved motifs (Motifs 1–15). (**Right**) The exon/intron (CDS/UTR) structures of the corresponding genes. Clade and subgenome assignment are indicated by colored bars.

Conserved-motif and gene-structure analyses revealed both broad conservation and clade-associated divergence within the *PgUGT* family (Figure 3 and Figure S1). A MEME analysis of the full-length sequences of all 212 PgUGT proteins identified 15 enriched amino acid motifs [45]. Members within the same phylogenetic group generally exhibited similar motif arrangements, whereas more pronounced differences were observed among clades.

The canonical PSPG box of plant family-1 UGTs is approximately 44 amino acids long. Motif 1 identified in the present analysis was 29 amino acids long and covered the conserved core region of the PSPG box involved primarily in the recognition of UDP-activated sugar donors [3,4,5]. This motif 1 was present in 208 of the 212 PgUGT proteins (98.1%), but was absent in four members (*PgUGT005*, *PgUGT131*, *PgUGT142*, and *PgUGT200*), all of which were confirmed as UGTs by Pfam domain and PSPG motif analyses (Figure S2; Table S1). Notably, these four variants retain the conserved histidine (His) residue characteristic of the PSPG motif, suggesting they may represent natural PSPG variants. Motifs 2–4 were likewise retained in most members, indicating broad conservation of the core regions associated with UGT catalytic function and sugar-donor recognition. By contrast, several non-core motifs showed clade- or subclade-associated distributions, and Clade V exhibited comparatively diverse motif combinations. Overall, the *PgUGT* family was characterized by strong conservation of PSPG-related core motifs accompanied by clade-associated variation in non-core regions.

Gene-structure analysis further revealed substantial variation among *PgUGT* members. Intron numbers ranged from 0 to 15, and 98 *PgUGT* genes were intronless. Most members of Clades I–III contained at least one intron, whereas intronless genes were more frequent in Clades IV and V, suggesting clade-associated differences in gene structure. Similar intron-poor patterns have been reported in other plant gene families [18,61]. Thus, the contrasting intron compositions provide additional evidence of structural differentiation among *PgUGT* clades.

### 3.4. WGD/Segmental Duplications Predominate in PgUGT Copy Retention and Expansion

**Figure 4:**
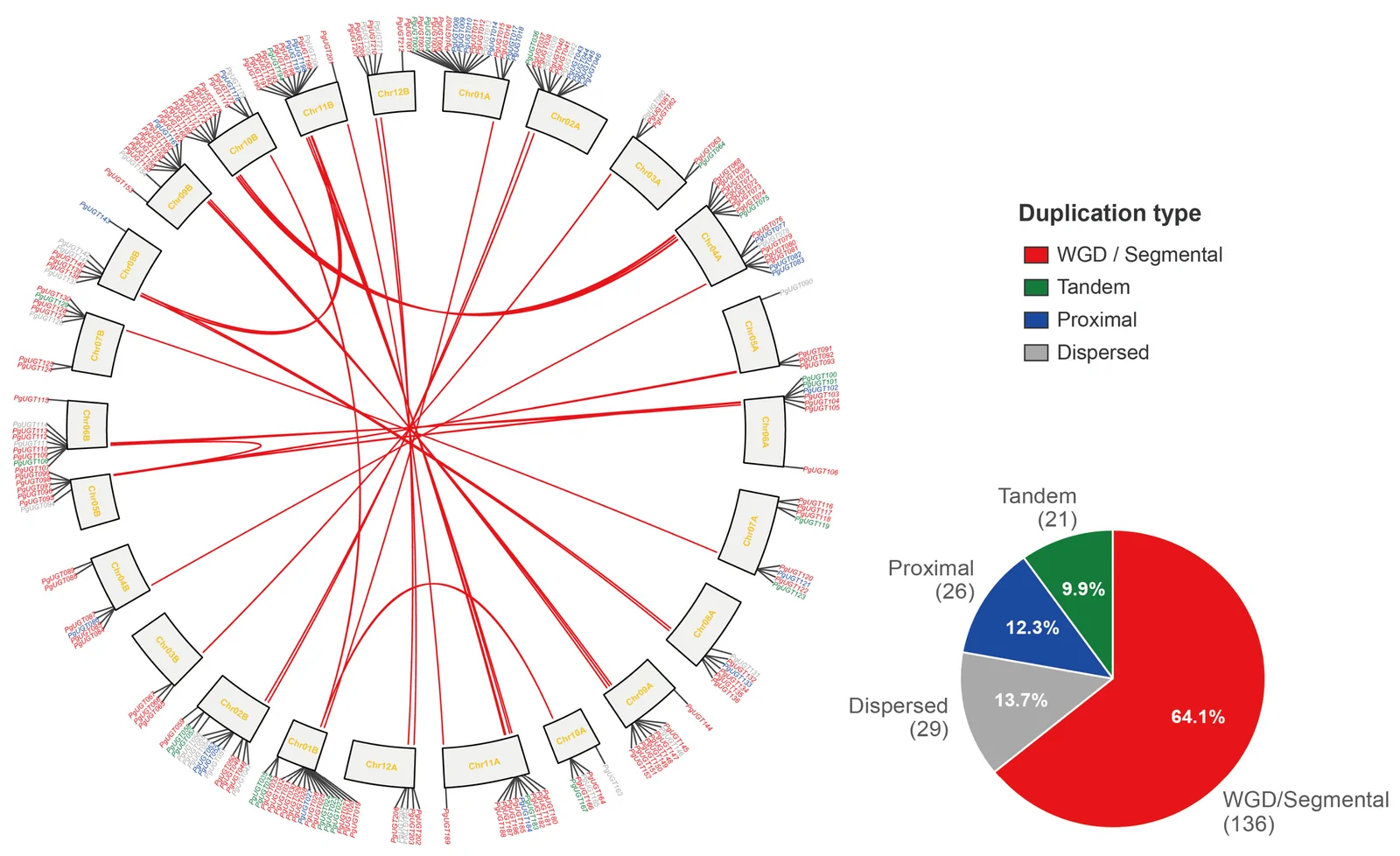
Collinearity and duplication types of the *PgUGT* family. (**Left**) Circos plot showing *PgUGT–PgUGT* collinear homologous pairs across the 24 chromosomes (red links denote WGD/segmental duplication), with gene names colored by duplication type. (**Right**) Donut chart illustrating the proportions of the four duplication types: WGD/segmental 136 (64.1%), dispersed 29 (13.7%), proximal 26 (12.3%), and tandem 21 (9.9%).

To further dissect the evolution of the *PgUGT* family, the 212 *PgUGT*s were classified into four duplication categories based on genome collinearity and the physical relationships among genes (Figure 4) [46,47,48]. Whole-genome duplication/segmental duplication (WGD/segmental duplication) was the predominant category, comprising 136 genes (64.2%), including 64 members from subgenome A and 72 from subgenome B. Dispersed, proximal, and tandem duplications accounted for 29 (13.7%), 26 (12.3%), and 21 (9.9%) members, respectively. These results indicate that WGD/segmental duplication was the principal source of *PgUGT* family expansion and copy retention, with broadly comparable contributions from the two subgenomes.

Collinearity analysis identified 43 unique *PgUGT*–*PgUGT* collinear gene pairs (encompassing 81 unique loci; Figure 4). Of these, 39 pairs (90.7%) connected subgenomes A and B across all five clades, whereas the remaining four pairs occurred within subgenome B. Among the 39 cross-subgenome pairs, 30 were located on correspondingly numbered homologous chromosome groups, whereas nine connected differently numbered A and B chromosome groups. These results demonstrate extensive retention of collinear *PgUGT* copies across the two subgenomes, accompanied by local deviations from strict one-to-one chromosomal correspondence.

A local Clade II gene array comprising *PgUGT021*–*PgUGT024* was also identified on Chr01/01B. Both *Ka* and *Ks* were zero for the pairwise comparisons among *PgUGT021*–*PgUGT023*, indicating that no nonsynonymous or synonymous substitutions were detected in the coding sequences analyzed. By contrast, the *Ks* value between *PgUGT024* and each of the other three genes was 1.343, indicating greater sequence divergence (Table S2). Together, these results show that the retention of WGD-derived homologs and subsequent local duplication jointly shaped the size and chromosomal organization of the *PgUGT* family.

### 3.5. Predominant Purifying Selection Across Duplicated PgUGTs

To assess selective pressure on the *PgUGT* family, *Ka* and *Ks* substitution rates were analyzed for homologous gene pairs. Valid *Ka*/*Ks* estimates were obtained for 442 pairs (Table S2), of which 440 (99.5%) had *Ka*/*Ks* < 1 and only two (0.5%) had *Ka*/*Ks* > 1. The ratios were concentrated in the lower intervals: 109 pairs (24.7%) had values below 0.2, 290 (65.6%) ranged from 0.2 to 0.5, and 41 (9.3%) ranged from 0.5 to 1.0. The family-wide mean and median *Ka*/*Ks* values were 0.298 and 0.254, respectively. These results indicate that the coding sequences of most *PgUGT* homologous pairs have been subject to purifying selection. The two pairs with *Ka*/*Ks* > 1 were *PgUGT017*/*PgUGT018* (1.459) and *PgUGT076*/*PgUGT078* (1.387), which exhibited low synonymous divergence (*Ks* = 0.0135 and 0.0034), where minor substitution stochasticity can inflate pairwise ratios (Table S2).

**Figure 5:**
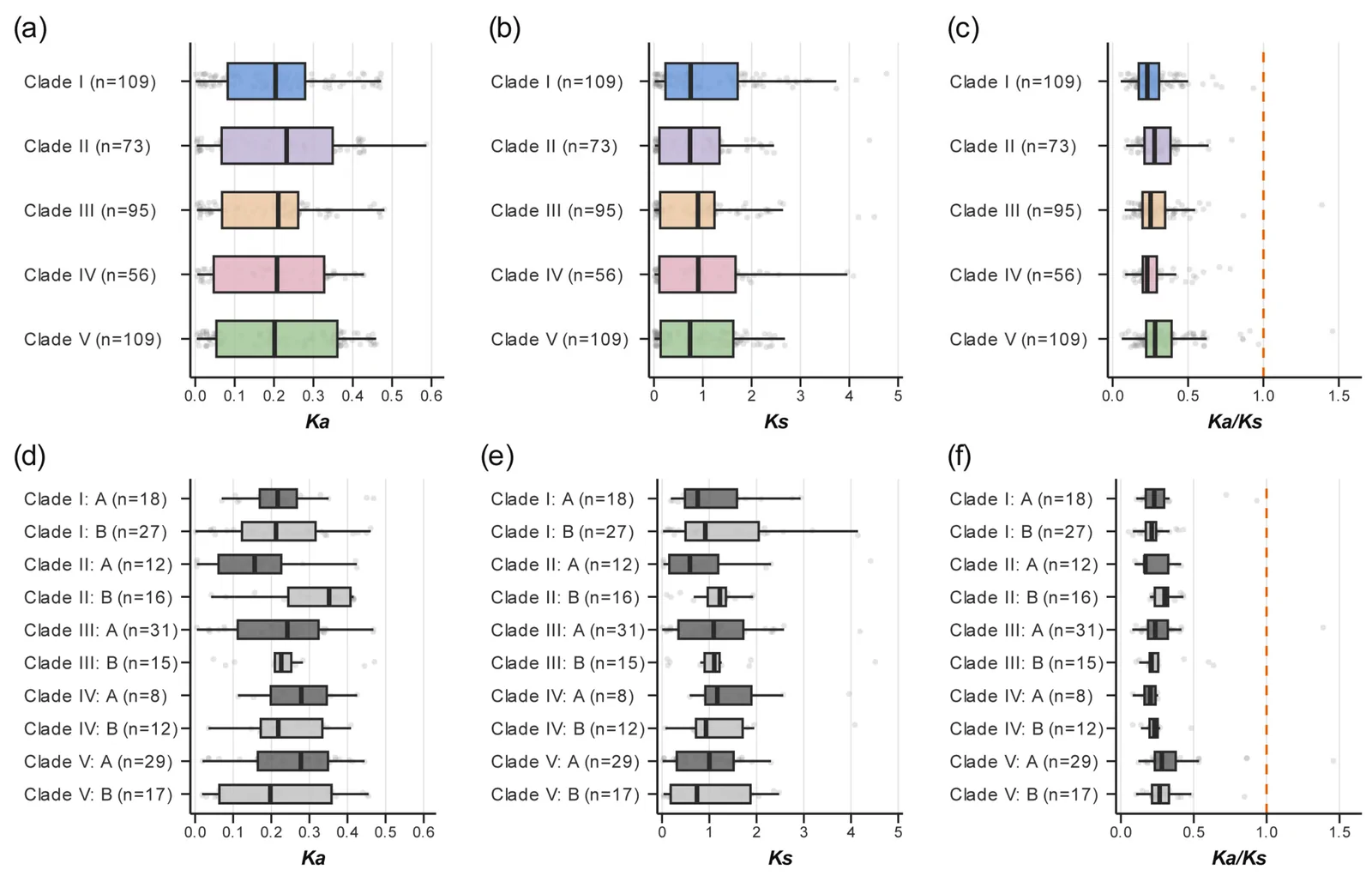
Selective pressure analysis of *PgUGT* homologous pairs. (**a**–**c**) Distributions of *Ka*, *Ks*, and *Ka*/*Ks* compared across the five clades (Clades I–V) based on 442 pairs with finite estimates (nine identical pairs recorded as NA). (**d**–**f**) Distributions of *Ka*, *Ks*, and *Ka*/*Ks* further compared among A/B subgenome pairings within clades. The orange dashed line marks *Ka*/*Ks* = 1.0.

The distributions of *Ka*, *Ks*, and *Ka*/*Ks* were subsequently compared among the five phylogenetic clades using all 442 pairs (Figure 5a–c). No significant differences were detected among clades for *Ka* or *Ks* (Kruskal–Wallis test, *p* = 0.632 and 0.881, respectively). To examine whether selective constraints differed between the two subgenomes within individual clades, A–A and B–B homologous pairs were compared separately in each clade (Figure 5d–f). These analyses included 185 within-subgenome pairs, comprising 98 A–A and 87 B–B pairs. The remaining 257 A–B pairs were retained in Figure 5a–c and Table S2 but were not assigned to either subgenome in the within-clade comparisons. Although the relative distributions of *Ka*, *Ks*, and *Ka*/*Ks* varied among clades, none of the A–A versus B–B comparisons remained significant after Benjamini–Hochberg correction of the two-sided Wilcoxon rank-sum tests (adjusted *p* > 0.05). Collectively, these results demonstrate widespread purifying selection and clade-associated variation in selective constraint, but do not support a consistent directional difference between the A and B subgenomes.

### 3.6. PgUGT Co-Expression Modules Show Organ- and Development-Associated Patterns

**Figure 6:**
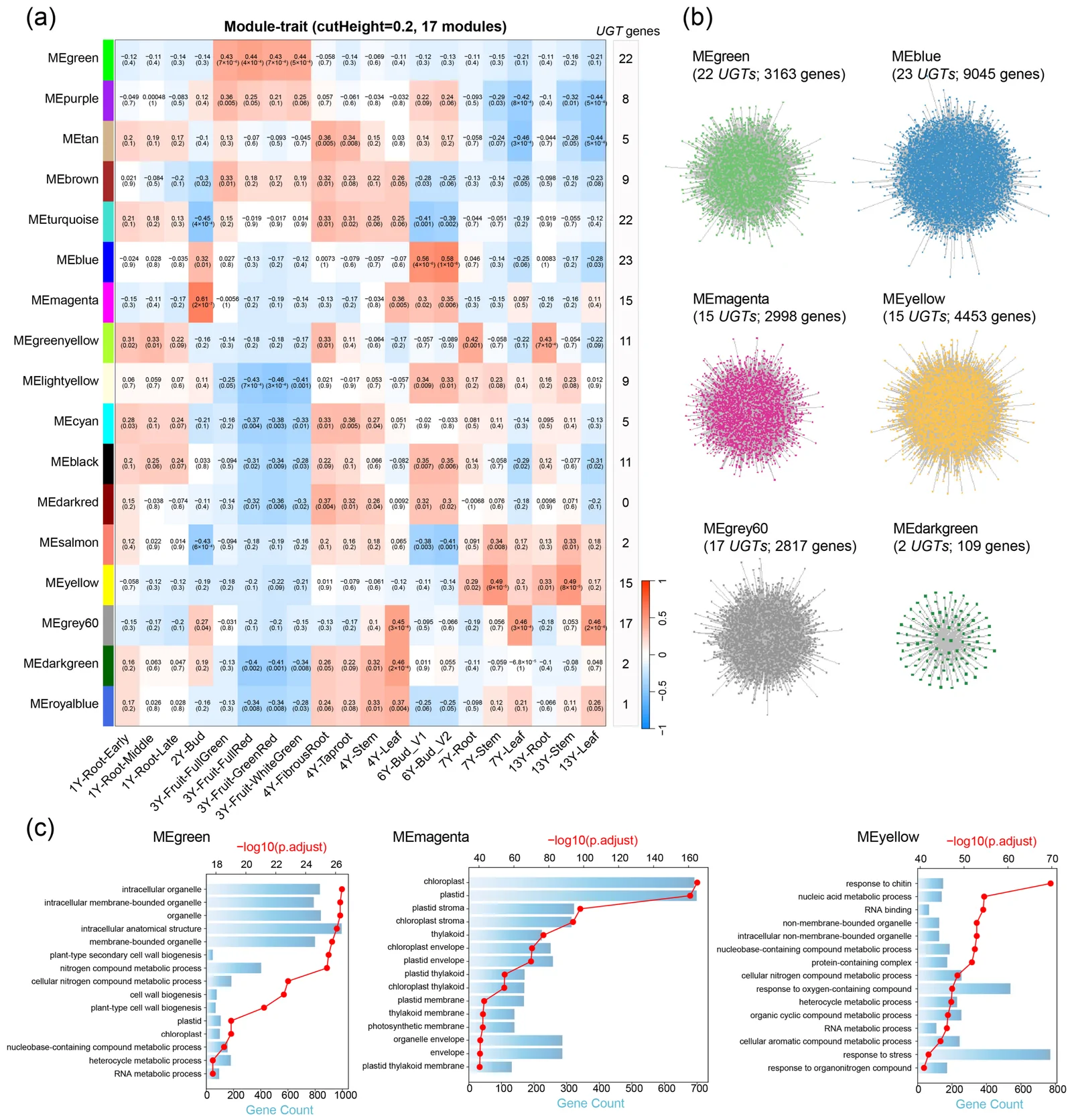
Weighted gene co-expression network analysis of developmental transcriptomes across 23 groups. (**a**) Correlations between module eigengenes and developmental/tissue traits; cells show Pearson’s correlation coefficients and Benjamini–Hochberg-adjusted *p* values, and the right column gives the number of *PgUGT*s per module. (**b**) Co-expression networks of representative modules containing *PgUGT*s. (**c**) Representative Gene Ontology enrichment summaries for MEgreen, MEmagenta, and MEyellow against the whole-genome annotation background (FDR < 0.05).

To characterize the expression features of *PgUGT*s during growth and development, we integrated publicly available RNA-seq data from the NCBI SRA [50] with in-house data generated in this study. The dataset comprised 23 tissue or developmental conditions, including root, storage root, bud, fruit, stem, and leaf samples from 1–4, 6–7, and 13-year-old plants. Additionally, fruit data covered four maturation stages from 3-year-old material (Figure S3; Table S3). Weighted gene co-expression network analysis (WGCNA) [55] partitioned the genes included in the analysis into 17 co-expression modules, 16 of which contained *PgUGT* members (Figure 6a). MEblue, MEturquoise, and MEgreen contained the largest numbers of *PgUGT*s, with 23, 22, and 22 members, respectively, whereas MEsalmon and MEroyalblue contained only two and one *PgUGT*, respectively. Module–trait correlation analysis showed that MEblue was positively correlated with 6-year-old bud samples, MEturquoise was negatively correlated with 2-year-old buds, and MEgreen was positively correlated with the different maturation stages of 3-year-old fruits. These results indicate that different *PgUGT* members were assigned to co-expression modules associated with distinct tissues or developmental stages.

Based on module–trait correlations, six representative modules containing *PgUGT* members were selected for co-expression network visualization (Figure 6b). Among these, MEgreen contained 22 *PgUGT*s and was positively correlated with the different maturation stages of 3-year-old fruits; MEblue contained 23 *PgUGT*s and was positively correlated with 6-year-old bud samples; and MEmagenta contained 15 *PgUGT*s and was positively correlated with 2-year-old buds. Among the modules associated with vegetative organs, MEyellow contained 15 *PgUGT*s and was positively correlated with 7- and 13-year-old stem tissues; MEgrey60 contained 17 *PgUGT*s and was positively correlated with leaf samples of different plant ages; and MEdarkgreen contained two *PgUGT*s and was positively correlated with 4-year-old leaves. These module–trait relationships show that *PgUGT* co-expression modules display differential association patterns across organs, plant ages, and developmental stages.

To characterize the functional context of the co-expression modules containing *PgUGT*s, Gene Ontology (GO) enrichment analysis was performed on the gene sets associated with the representative modules (Figure 6c). The MEgreen module, which was associated with the different maturation stages of 3-year-old fruits, was significantly enriched in terms related to the cell wall, intracellular organelles, and intracellular anatomical structures. The MEmagenta module, associated with 2-year-old buds, was mainly enriched in photosynthesis-related terms such as chloroplast, plastid, and thylakoid. The MEyellow module, associated with 7- and 13-year-old stem tissues, was significantly enriched in functional terms including response to chitin, nucleic acid metabolism, and RNA binding. Taken together, these results suggest that *PgUGT*-containing modules exhibit distinct tissue associations and functional contexts.

### 3.7. Composition and Member-Level Variation in Promoter Cis-Acting Elements

**Figure 7:**
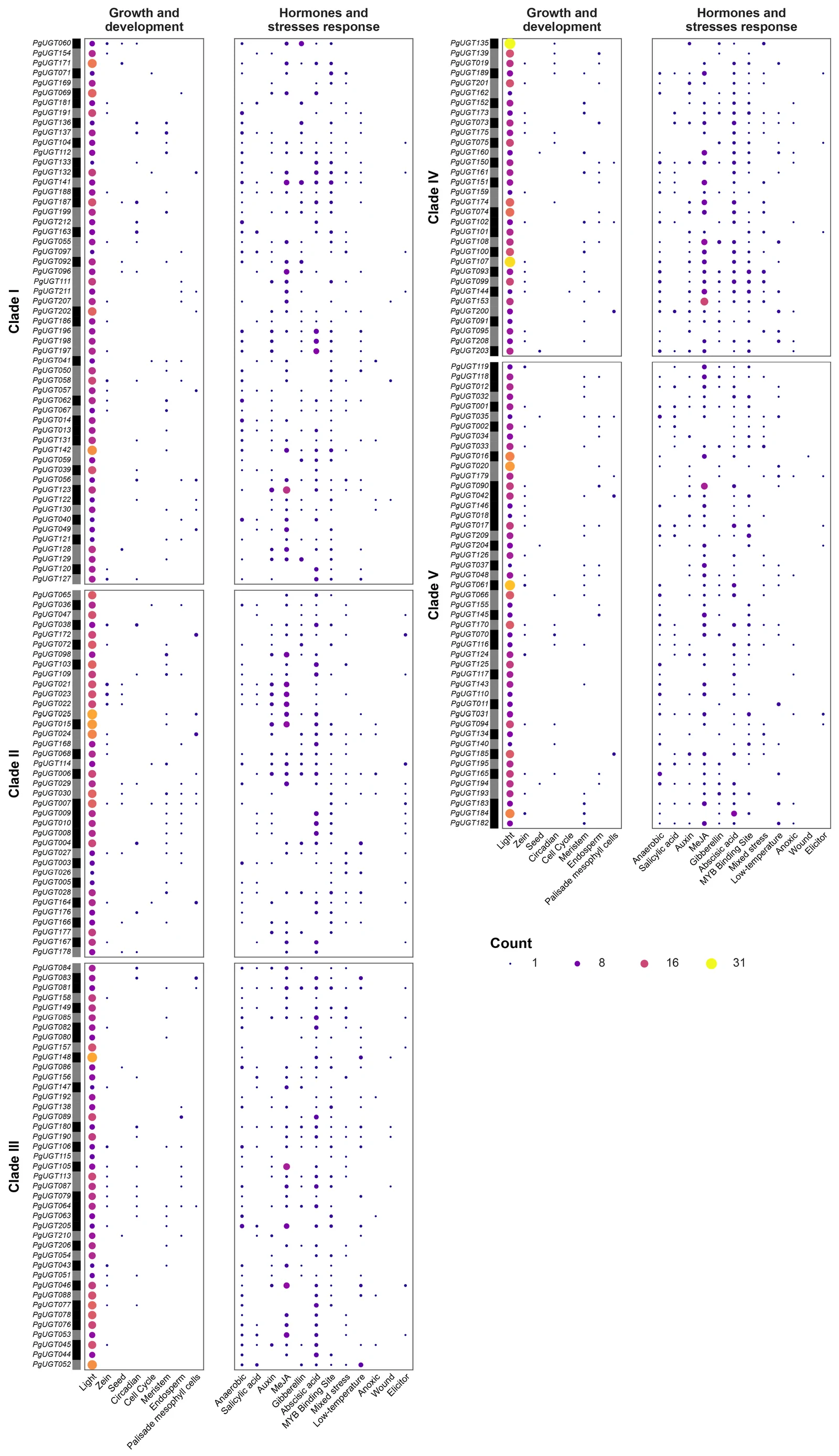
Predicted cis-element composition of 212 *PgUGT* promoters. Bubble size and color encode the curated number of records in each category within the 2-kb upstream region (5371 records in total, Table S5).

To characterize the potential upstream regulatory features of *PgUGT* genes, we extracted the 2-kb genomic sequence upstream of the annotated start codon of all 212 *PgUGT*s as the candidate promoter region and predicted cis-acting elements using the PlantCARE database [59]. After functional annotation curation and standardization of category names, a total of 5371 predicted element records were obtained and assigned to the 20 categories shown in Figure 7, covering elements related to growth and development, hormone responses, and environmental stress (Figure 7; Table S5). Among these, light-responsive elements were the most abundant, with 2668 records distributed across all 212 promoters. These were followed by methyl jasmonate (MeJA)-responsive elements, with 570 records distributed across 144 genes; abscisic acid (ABA)-responsive elements, with 453 records across 166 genes; MYB binding sites, with 277 records across 156 genes; and anaerobic induction elements, with 246 records across 141 genes. In addition, auxin- and gibberellin-responsive elements were present in the promoters of 118 and 107 *PgUGT*s, respectively. These results suggest that *PgUGT* promoters may be rich in light-responsive elements and may harbor a broad distribution of candidate regulatory sites associated with hormone and environmental stress signaling.

Comparison of *PgUGT*s grouped by clade indicated that all five clades contained multiple types of cis-acting elements, but that the number and combination of element categories tended to vary mainly among individual gene members. Light-responsive elements were present in all 212 promoters, with *PgUGT135* (Clade IV) and *PgUGT107* (Clade IV) showing relatively high numbers of records, at 31 and 29, respectively, followed by *PgUGT061* (Clade V), *PgUGT025* (Clade II), and *PgUGT148* (Clade III), with 27, 25, and 25 records, respectively. The distribution of MeJA-responsive elements appeared more variable: *PgUGT153* (Clade IV) contained 16 records, *PgUGT123* (Clade I) contained 14, and *PgUGT105* (Clade III) and *PgUGT090* (Clade V) each contained 12, whereas no elements of this category were detected in 68 promoters. For ABA-responsive elements, *PgUGT197* (Clade I) and *PgUGT184* (Clade V) showed relatively high numbers of records, with nine each, whereas for MYB binding sites, *PgUGT209* (Clade V), *PgUGT141* (Clade I), and *PgUGT093* (Clade IV) showed the highest numbers, with five each. Elements associated with mixed stress, low temperature, anaerobic induction, and elicitor responses were distributed across 86, 79, 141, and 31 *PgUGT* promoters, respectively, further reflecting differences in element combinations among members. Overall, the cis-acting elements of *PgUGT* promoters showed member-specific compositional patterns, with differences between the A and B subgenomes also noted in some members.

### 3.8. Transcriptional Responses to Biotic Stress

**Figure 8:**
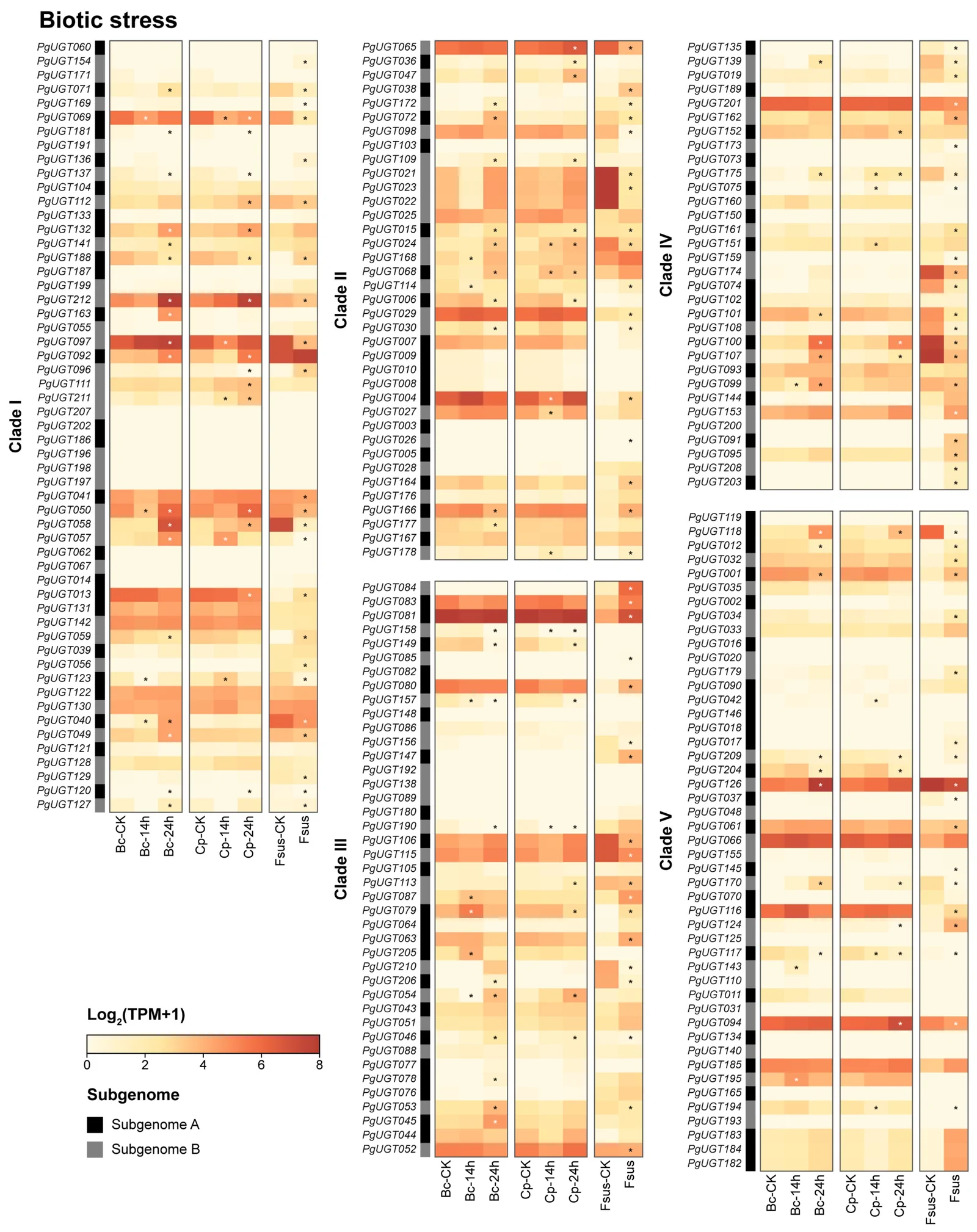
Biotic-stress expression of 212 *PgUGT*s. The heatmap shows log2(TPM + 1)-transformed expression values across *Botrytis cinerea* (*Bc*), *Colletotrichum panacicola* (*Cp*), and *Fusarium solani* (*Fsus*) condition groups. Treatment–control comparisons were matched within source projects. Asterisks indicate significant differential expression (FDR padj < 0.05 and |log_2_FC| ≥ 1.0).

To assess the transcriptional responses of the *PgUGT* family to pathogen infection, we analyzed the expression changes of all 212 family members under three ginseng pathogen treatments, comprising the leaf pathogens *Botrytis cinerea* (*Bc*) and *Colletotrichum panacicola* (*Cp*) and the root pathogen *Fusarium solani* (*Fsus*) (Figure 8; Tables S6 and S7). Given that the leaf and root pathogens correspond to different tissue contexts, the two leaf pathogen treatments were compared first. The number of candidate *PgUGT*s detected at 24 h exceeded that at 14 h for both the *Bc* and *Cp* treatments. Under the *Bc* treatment, 6 upregulated and 8 downregulated genes were detected at 14 h, increasing to 34 and 18, respectively, at 24 h; under the *Cp* treatment, 10 upregulated and 8 downregulated genes were detected at 14 h, increasing to 25 and 17, respectively, at 24 h. These results indicate that the number of candidate *PgUGT* members increased with sampling time under both leaf pathogen treatments.

Further comparison of the responses at the two sampling time points for the same pathogen showed that, under the *Cp* treatment, *PgUGT024* and *PgUGT068* (Clade II), *PgUGT175* (Clade IV), and *PgUGT211* (Clade I) were upregulated at both 14 h and 24 h, whereas *PgUGT069* (Clade I), *PgUGT117* (Clade V), and *PgUGT158* and *PgUGT190* (Clade III) were downregulated at both time points. These eight genes constituted the complete intersection of genes differentially expressed in the same direction at 14 h and 24 h under the *Cp* treatment, indicating that they maintained a consistent response direction across the two sampling time points.

At the same 24-h sampling point, the *Bc* and *Cp* treatments shared 17 upregulated and 11 downregulated *PgUGT*s, indicating overlap between the transcriptional responses induced by the two leaf pathogens. Among these, *PgUGT024*, *PgUGT068* (Clade II), and *PgUGT175* (Clade IV) were upregulated at both 14 h and 24 h under the *Cp* treatment and maintained the same response direction at 24 h under the *Bc* treatment, whereas *PgUGT117* (Clade V), *PgUGT158*, and *PgUGT190* (Clade III) were downregulated in all three comparisons (Table S7). These genes showed consistent expression changes across the sampled leaf pathogens and time points and are therefore priority candidates for subsequent functional testing in these contexts.

Under the root pathogen *Fsus* treatment, 40 upregulated and 58 downregulated *PgUGT*s were detected, involving multiple clades and different chromosomal regions. Notably, the adjacent genes *PgUGT084*, *PgUGT085* and *PgUGT087* (Clade III) on Chr14/04B were consistently upregulated, whereas the local array *PgUGT021*, *PgUGT023* and *PgUGT024* (Clade II) on Chr01/01B were markedly downregulated. In addition, *PgUGT153* (Clade IV) and *PgUGT161*–*PgUGT162* (Clade IV), located in other chromosomal regions, were also upregulated. These results indicate that the transcriptional response induced by the *Fsus* treatment involves both specific members distributed across different clades and a concentration within certain local chromosomal gene clusters; adjacent *PgUGT* copies within the same region showed a consistent response direction, providing a basis for identifying candidate genes associated with root pathogen responses.

### 3.9. Expression Responses to Abiotic Stress and Hormone and Chemical Treatments

**Figure 9:**
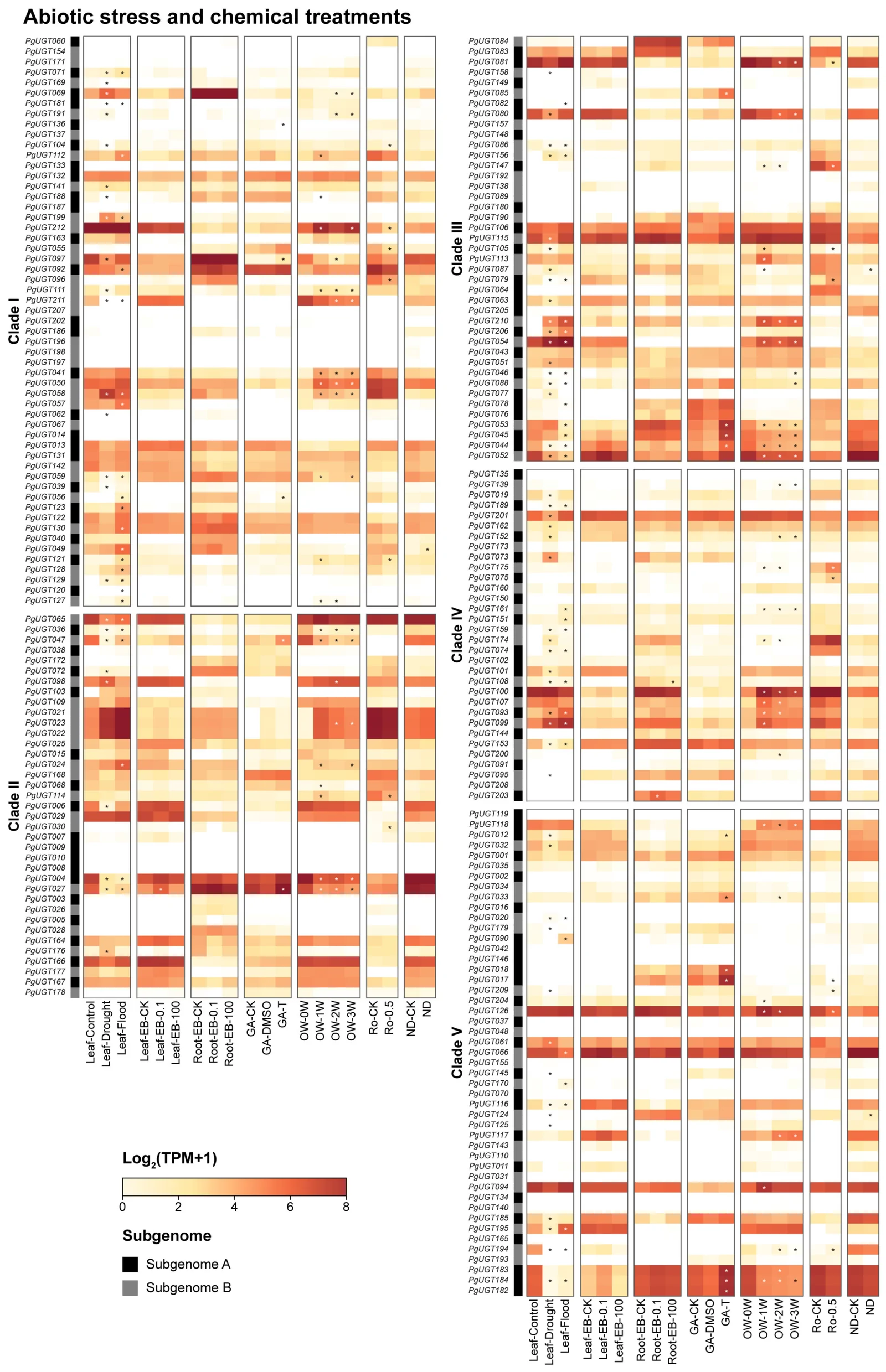
Expression profiles of 212 *PgUGT* genes under abiotic stress, hormone, and chemical treatments. The heatmap shows log_2_(TPM + 1)-transformed expression values. Leaf-Control, untreated leaf control paired with drought and flooding treatments; Leaf-Drought, leaf drought treatment; Leaf-Flood, leaf flooding treatment; Leaf-EB-CK, untreated leaf control for endo-borneol (EB); Leaf-EB-0.1 and Leaf-EB-100, leaf samples after 0.1 and 100 mg/L EB treatments, respectively; Root-EB-CK, untreated root control for EB; Root-EB-0.1 and Root-EB-100, root samples after 0.1 and 100 mg/L EB treatments, respectively; GA-CK, untreated control paired with gibberellic acid treatment; GA-DMSO, dimethyl sulfoxide solvent control; GA-T, gibberellic acid treatment; OW-0W, OW-1W, OW-2W, and OW-3W, continuous overwatering samples collected at 0, 1, 2, and 3 weeks, respectively; Ro-CK, untreated control paired with exogenous ginsenoside Ro; Ro-0.5, 0.5 mg/L exogenous ginsenoside Ro treatment; ND-CK, normal-nitrogen control; ND, nitrogen-deficiency treatment. Asterisks indicate significant differential expression (FDR padj < 0.05 and |log_2_FC| ≥ 1.0).

To assess the transcriptional responses of the *PgUGT* family to environmental changes and exogenous chemical signals, we analyzed the expression changes of all 212 family members under drought, flooding, continuous overwatering (OW), nitrogen deficiency (ND), gibberellic acid (GA), endo-borneol (EB), and exogenous ginsenoside Ro treatments (Figure 9; Tables S6 and S7).

Among the water-related treatments, 32 upregulated and 41 downregulated *PgUGT*s were detected under drought, and 31 upregulated and 24 downregulated genes were detected under flooding, indicating that both water deficit and water excess can elicit transcriptional changes in multiple family members. Because drought and water excess represent distinct water states, the following analysis focuses on integrating the response features of the flooding and continuous overwatering (OW) treatments. Relative to the 0-week baseline, 26, 21, and 15 upregulated genes and 12, 19, and 18 downregulated genes were detected at 1, 2, and 3 weeks of OW treatment, respectively; among these, nine *PgUGT*s were upregulated at all three time points, and 8 were downregulated at all three. Further comparison with the flooding treatment showed that *PgUGT054* (Clade III), *PgUGT058* (Clade I), *PgUGT161* (Clade IV) and *PgUGT210* (Clade III) were upregulated under flooding and at all three OW time points, whereas *PgUGT004*, *PgUGT027* and *PgUGT036* (Clade II), *PgUGT044* (Clade III), *PgUGT047* (Clade II), *PgUGT052*–*PgUGT053* (Clade III), and *PgUGT184* (Clade V) were downregulated in all four comparisons (Table S7). These genes maintained a consistent response direction across the water-excess treatments and successive sampling time points, constituting a repeated-response candidate set. These candidates were distributed across multiple clades, indicating that water-stress responses involve phylogenetically diverse *PgUGT* lineages.

Among the hormone treatments, GA elicited predominantly upregulated responses relative to both controls: 22 upregulated and three downregulated genes were detected relative to the GA-matched control (GA-CK), and 25 upregulated and six downregulated genes were detected relative to the dimethyl sulfoxide (DMSO) solvent control. The two comparisons shared 15 upregulated genes, including *PgUGT012* (Clade V), *PgUGT017*–*PgUGT018* (Clade V), *PgUGT027* (Clade II), *PgUGT033* (Clade V), *PgUGT044*–*PgUGT045* (Clade III), *PgUGT047* (Clade II), *PgUGT053* (Clade III), *PgUGT056* (Clade I), *PgUGT085* (Clade III), *PgUGT097* (Clade I), and *PgUGT182*–*PgUGT184* (Clade V), as well as one consistently downregulated gene, *PgUGT136* (Clade I). Because these 16 genes were detected under both control settings, they constitute a GA-responsive candidate set supported by relatively consistent evidence.

Transcriptional responses of *PgUGT*s to EB treatment differed by tissue type and concentration. In roots, only *PgUGT203* (Clade IV) was significantly upregulated at 0.1 mg/L EB, whereas only *PgUGT108* (Clade IV) was significantly downregulated at 100 mg/L EB. In leaves, only *PgUGT027* (Clade II) was significantly upregulated at 0.1 mg/L EB, and no *PgUGT* met the significance threshold at 100 mg/L EB. Treatment with exogenous ginsenoside Ro resulted in the upregulation of five *PgUGT*s (*PgUGT030*, *PgUGT075*, *PgUGT079*, *PgUGT175*, and *PgUGT194*) and the downregulation of 12 genes (*PgUGT017*, *PgUGT055*, *PgUGT081*, *PgUGT096*, *PgUGT104*, *PgUGT105*, *PgUGT114*, *PgUGT121*, *PgUGT126*, *PgUGT147*, *PgUGT209*, and *PgUGT212*) (Table S7). In addition, two *PgUGT*s (*PgUGT049* and *PgUGT124*) were upregulated, and one (*PgUGT087*) was downregulated under nitrogen deficiency. These results suggest that *PgUGT* responses to exogenous chemical signals depend on the specific agent, its concentration, and the tissue type.

Overall, the transcriptional responses of the *PgUGT* family to water-status changes and to hormone and exogenous chemical treatments were concentrated mainly in specific members and certain local gene clusters rather than reflecting synchronized changes across entire clades. Multiple *PgUGT*s showed consistent expression trends across flooding and continuous overwatering treatments, as well as under the two GA control conditions, suggesting a subset of candidate genes potentially involved in common or overlapping stress and hormone responses.

## 4. Discussion

Allopolyploidization provides duplicated genetic resources that can be retained, lost, or diversified during genome evolution. In this study, 212 *PgUGT*s were systematically identified using the ginseng T2T reference genome and analyzed comprehensively with respect to phylogeny, gene structure, duplication modes, selection pressure, expression regulation, and stress responses. Subgenomes A and B contained 104 and 108 members, respectively, and the five major clades were distributed at a near 1:1 ratio between the subgenomes, showing extensive reciprocal retention. WGD/segmental duplication was the principal source of family expansion, whereas tandem duplication contributed to the formation of local gene clusters. The vast majority of the gene pairs analyzed had *Ka*/*Ks* < 1, indicating that *PgUGT*s are predominantly subject to purifying selection, although selective constraints varied among phylogenetic clades and no consistent directional difference was detected between the A and B subgenomes. Co-expression analysis revealed that *PgUGT* members were associated with the development of buds, stems, leaves, and fruits. Promoter analysis together with the analyses of biotic and abiotic stress responses further supported the potentially diverse functional roles of *PgUGT*s. Taken together, this study provides new insights into the evolution of the UGT family in allopolyploid medicinal plants and provides a foundation for prioritizing candidate *PgUGT* genes for subsequent functional investigation.

Local differences in copy number among homologous chromosomes suggest that family evolution involved both ancestral copy preservation and subsequent lineage-specific expansion at local loci. The 39 cross-subgenome collinear homoeolog pairs are retained across all five clades, and pairwise expression comparisons between subgenome partners revealed no global subgenome expression dominance, potentially indicating balanced functional retention between subgenomes A and B [13]. At the level of sequence architecture, the core PSPG motif was retained in nearly all *PgUGT*s, consistent with strong selective constraint on the sugar-donor recognition region, whereas distal regions were more variable. By comparison, non-core motifs and intron structures diverged more markedly among clades, with intronless members being more frequent in Clades IV and V, reflecting clade-associated differences in gene-structure evolution [18,61]. Considering the *Ka*/*Ks* distribution together with the copy-number differences between homologous chromosomes, the *PgUGT* family displays an evolutionary pattern of overall balanced retention combined with local divergence; comparable mosaic patterns of divergence have also been reported in other allopolyploid plants [62]. This pattern suggests that the family did not undergo genome-wide replacement by one dominant subgenome after polyploidization; rather, differential retention and local expansion may have contributed to lineage-specific functional reorganization while maintaining overall family dosage.

Although duplicated genes often retain similar coding sequences, their evolutionary fate is frequently determined by changes in expression regulation. The integration of developmental transcriptomes, co-expression networks, and promoter analyses suggested potential regulatory divergence among *PgUGT* members. Among the 16 UGT-containing modules, the association of MEgreen with fruit maturation and of MEmagenta with bud development indicates that different members have been incorporated into distinct developmental regulatory networks. Differences in promoter cis-acting element composition provide further evidence for such regulatory divergence: the heterogeneous distribution of GA-, ABA-, and stress-responsive elements across the promoters of different members points to marked divergence in their transcriptional regulatory landscapes. Notably, adjacent members arranged in tandem, such as *PgUGT021*–*PgUGT023* and *PgUGT182*–*PgUGT184*, showed coordinated expression under particular treatments, consistent with the possibility that a locally shared regulatory environment contributes to this coordinated response [63,64]. However, the decoupling of expression among members of the same gene cluster under other conditions is also consistent with regulatory rearrangement and functional redistribution after tandem duplication. This pattern of local coordination combined with global divergence suggests that duplicated genes can gradually establish partly independent cis-regulatory landscapes while retaining some of their original regulatory associations.

The transcriptional responses of the *PgUGT* family across the various stress treatments showed pronounced member specificity and concentration within local gene clusters, rather than global changes across entire clades or subgenomes. As core enzymes of specialized metabolism, UGTs may show differential expression under stress that reflects condition-dependent allocation of metabolic resources. This interpretation is supported by the repeated upregulation of *PgUGT024* and *PgUGT175* alongside the downregulation of *PgUGT149* and *PgUGT158* under leaf-pathogen treatments, and by the opposite patterns displayed by the adjacent gene clusters on Chr14/04B and Chr01/01B under the root-pathogen *Fsus* treatment. Notably, certain members such as *PgUGT211* showed opposite regulatory directions between biotic-stress and drought/flooding treatments, indicating that the same UGT may participate differently in different signaling contexts. A comparable situation has been characterized for UGT76B1 in *A. thaliana*, which glycosylates three chemically distinct immune signaling molecules—salicylic acid, ILA, and NHP—and thereby acts differently in different defense contexts [65,66].

Based on the convergent indications from multiple data layers, future studies could prioritize *PgUGT024* and *PgUGT175* (leaf-pathogen responses), *PgUGT021*–*PgUGT023* (a shift in response between pathogen and water signals), and *PgUGT182*–*PgUGT184* (opposing response patterns). Functional validation could be pursued using CRISPR/Cas9 or hairy-root systems, combined with targeted metabolomics and enzyme-activity assays to identify their glycosyl acceptors and reaction products, and complemented by single-cell or spatial transcriptomics to resolve their cell-type and tissue-region specificity [67]. In summary, this study establishes a subgenome-resolved analytical framework for the ginseng UGT family and delineates its overall characteristics, including the emergence of member-specific and condition-dependent regulatory divergence alongside the maintenance of sequence conservation. These findings provide a case study for research on the evolution of specialized-metabolism-related gene families in allopolyploid medicinal plants and offer a prioritized set of candidate genes for subsequent functional characterization.

### Limitations of the Study

Several limitations should be considered. First, phylogenetic and collinearity analyses were confined to the allotetraploid *P. ginseng* genome; cross-species phylogenomic validation with diploid *Panax* relatives and outgroups would help refine the timing of diversification relative to allotetraploidization. Second, despite in silico assessment of the 212 candidate gene models (Table S1), automated annotation may retain some uncertainty in gene boundaries, alternative splicing, and pseudogene models. Third, the transcriptomic atlas combines newly sequenced and public RNA-seq data, and platform- or cultivar-related variation cannot be fully excluded. Fourth, PlantCARE-based promoter analysis is descriptive, identifying motif occurrences rather than verified transcription factor binding. Finally, the regulatory and functional interpretations, including subgenome divergence and prioritized PgUGTs, are computational and exploratory and would benefit from future biochemical and in vivo validation.

## 5. Conclusions

This study provides a comprehensive, subgenome-resolved investigation of the UDP-glycosyltransferase superfamily in allotetraploid *P. ginseng* based on a telomere-to-telomere assembly. The 212 identified *PgUGT* candidates display strong sequence conservation across core catalytic PSPG motifs alongside substantial clade-associated divergence in non-core architectures. Whole-genome duplication/segmental duplication predominated in shaping family expansion, maintaining balanced copy retention across subgenomes A and B under pervasive purifying selection. Transcriptomic and promoter analyses revealed pronounced subgenome expression divergence and member-specific, condition-dependent stress responsiveness. By systematically integrating candidate gene model quality metrics and explicitly delineating computational boundaries, this work establishes a prioritized genomic resource for functional characterization of ginsenoside biosynthetic enzymes and molecular breeding in ginseng.

## Data Availability

Transcriptome data generated in this study have been deposited in the National Genomics Data Center (NGDC) under accession number CRA044575. Public RNA-seq datasets reused in this study were obtained from the NCBI Sequence Read Archive (SRA), and their accession numbers are provided in Table S4. Genome-wide condition-level mean TPM matrices for all 77,266 annotated genes across 23 growth and development conditions and 28 biotic stress, abiotic stress, hormone, and chemical treatment conditions are available from figshare at https://doi.org/10.6084/m9.figshare.33137375. The *PgUGT*-specific TPM matrix and sample metadata are provided in Tables S3 and S4, respectively. Promoter cis-acting element records are provided in Table S5. Treatment–control summaries and candidate *PgUGT* responses are provided in Tables S6 and S7.

## Supplementary Materials

The following supporting information can be downloaded at https://www.mdpi.com/article/10.3390/genes17091140/s1. Figure S1. Sequence logos of the 15 conserved motifs. Figure S2. Validation of selected *PgUGT* candidates. Figure S3. Expression heatmap of the 212 *PgUGT*s across 23 growth and development conditions. Table S1. Molecular characteristics, clade assignments, duplication types, PF00201 domain evidence, PSPG box features, and the expression support of the 212 *PgUGT*s. Table S2. *Ka*, *Ks*, and *Ka*/*Ks* estimates for the 442 valid homologous gene pairs (with nine identical pairs recorded as NA). Table S3. TPM profiles of 212 *PgUGT* genes across 23 growth/development and 28 stress/treatment condition groups. Table S4. Metadata for 157 RNA-seq samples mapped to 51 condition-level profiles. Table S5. Curated cis-acting element records from the 2000-bp promoters of 212 *PgUGT* genes. Table S6. Summary of 18 within-project treatment–control comparisons for *PgUGT* expression responses. Table S7. Significant *PgUGT* responses across the 18 within-project treatment–control comparisons.

## Abbreviations

The following abbreviations are used in this manuscript:

ABA	Abscisic acid
ABA-GE	Abscisic acid glucose ester
*Bc*	*Botrytis cinerea*
Cd	Cadmium
CDS	Coding sequence
*Cp*	*Colletotrichum panacicola*
D3G	Deoxynivalenol-3-O-glucoside
DMSO	Dimethyl sulfoxide
DON	Deoxynivalenol
EB	Endo-borneol
FHB	Fusarium head blight
FR	Fine root
*Fsus*	*Fusarium solani*
GA	Gibberellic acid
GFF3	General Feature Format version 3
GO	Gene Ontology
GRAVY	Grand average of hydropathicity
IAA	Indole-3-acetic acid
IBA	Indole-3-butyric acid
ILA	Isoleucic acid
*Ka*	Nonsynonymous substitution rate
*Ks*	Synonymous substitution rate
MeJA	Methyl jasmonate
MEME	Multiple EM for Motif Elicitation
ML	Maximum likelihood
NCBI	National Center for Biotechnology Information
ND	Nitrogen deficiency
NGDC	National Genomics Data Center
NHP	N-hydroxypipecolic acid
OW	Overwatering
PR	Primary root
PSPG	Plant secondary product glycosyltransferase
RNA-seq	RNA sequencing
SRA	Sequence Read Archive
T2T	Telomere-to-telomere
TPM	Transcripts per million
UDP	Uridine diphosphate
UGT	UDP-glycosyltransferase
UTR	Untranslated region
WGCNA	Weighted gene co-expression network analysis
WGD	Whole-genome duplication
