# Supplementary material for: Subgenome-Resolved Analysis and Regulatory Divergence of UDP-Glycosyltransferases in Allotetraploid Panax ginseng
Source: Genes (Basel). 2026 Sep 17;17(9):1140. doi: 10.3390/genes17091140 (PMC13606172; doi:10.3390/genes17091140)
Supplement: Supplementary file 1 [file genes-17-01140-s001.zip › Supplementary_Figures.pdf]

Supplementary Figures

Subgenome-Resolved Analysis and Regulatory Divergence of UDP-Glycosyltransferases in Allotetraploid *Panax ginseng*

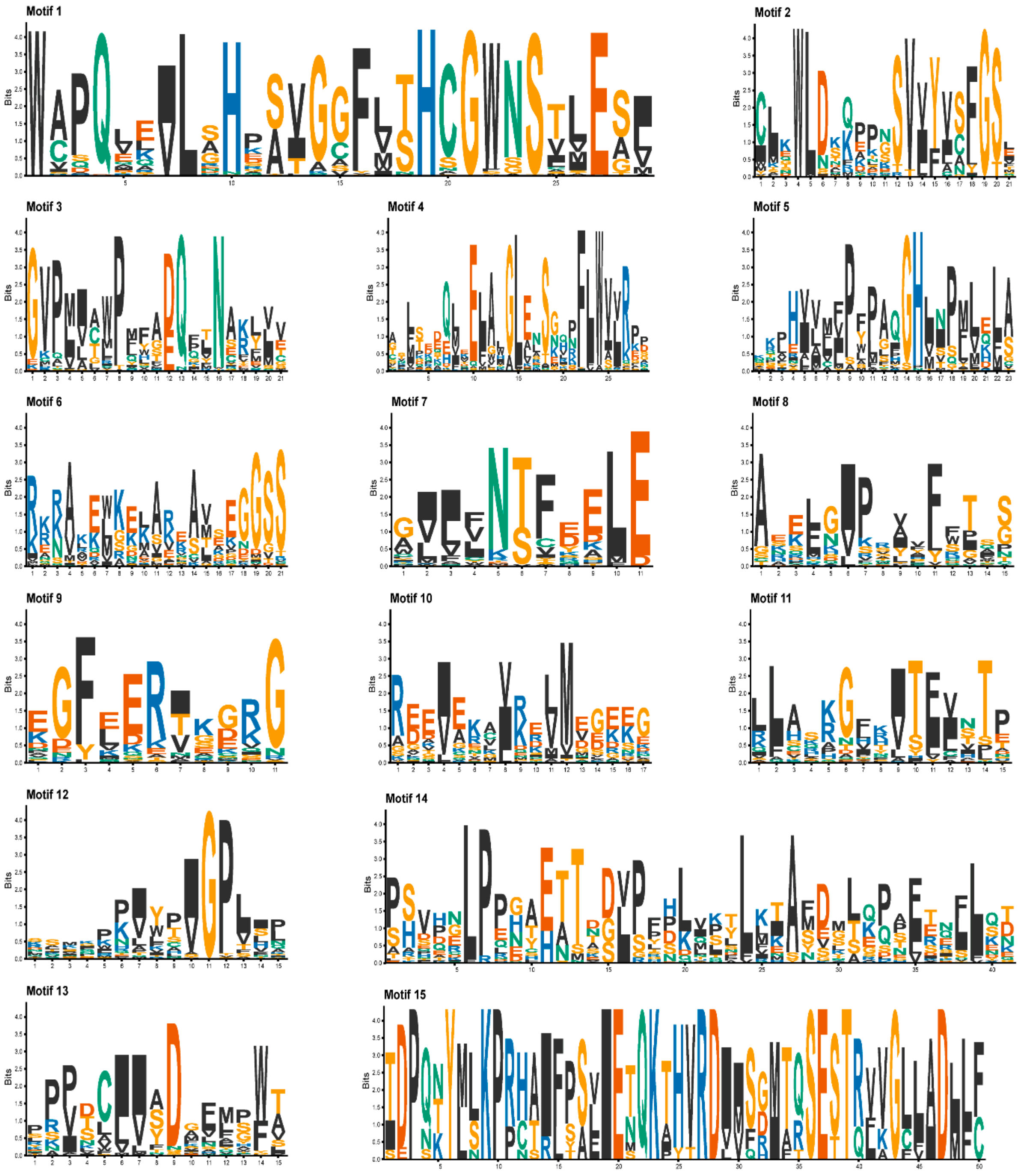

Figure S1. Sequence logos of the 15 conserved PgUGT motifs. Letter height represents amino-acid conservation at each position. Motif 1 corresponds to the plant secondary product glycosyltransferase (PSPG) box.

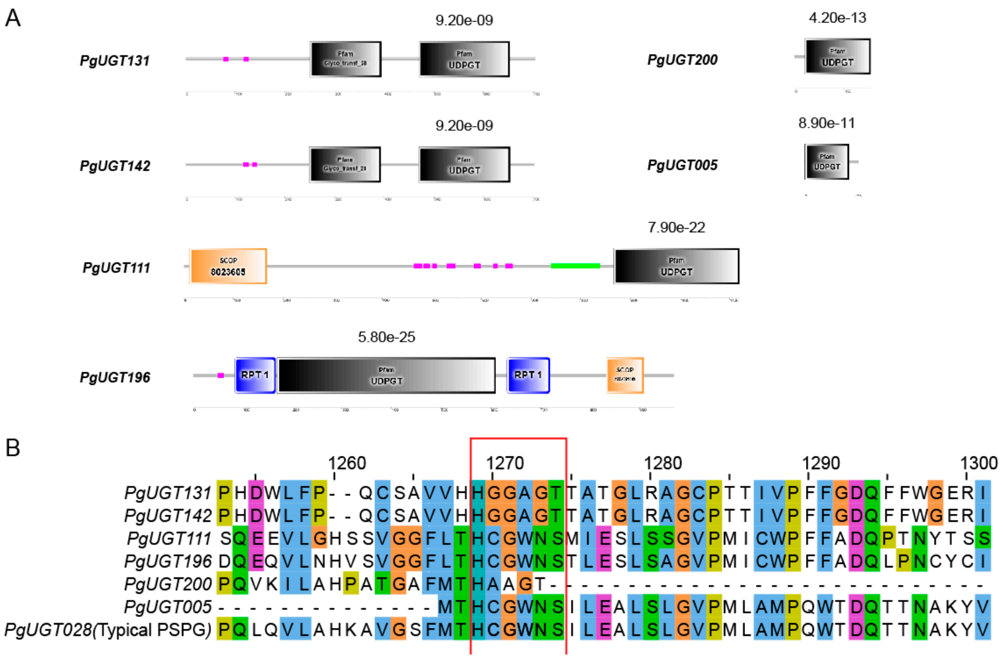

Figure S2. Validation of selected PgUGT candidates. (A) Pfam conserved domain identification of the six selected PgUGT genes; (B) Multiple sequence alignment of representative PgUGT proteins showing conserved PSPG motif.

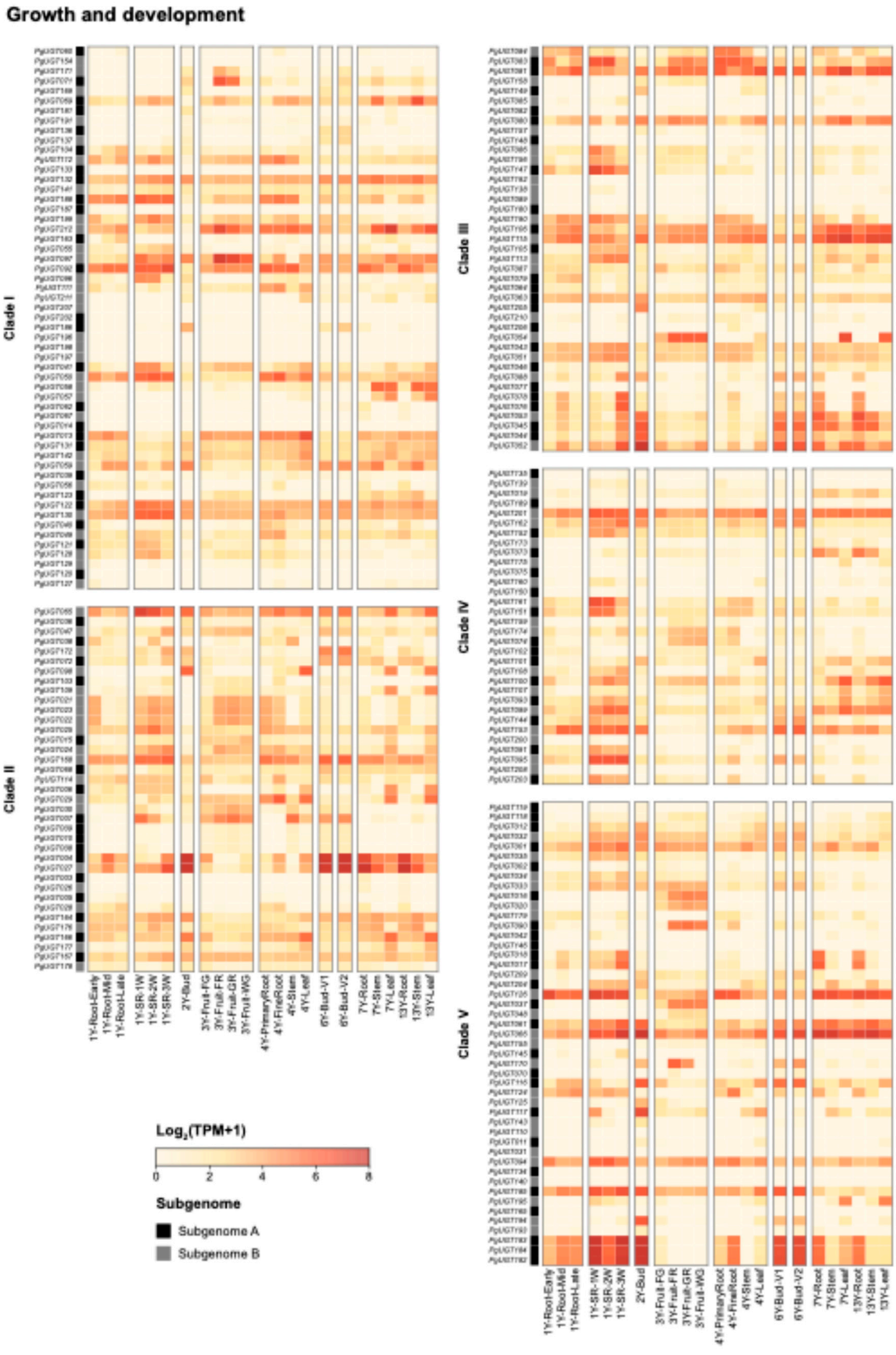

Figure S3. Developmental and tissue expression of 212 PgUGTs across 23 condition groups. In the sample labels, SR denotes storage root; FG, FR, GR and WG denote full-green, full-red, green-red and white-green fruit stages, respectively; FineRoot and PrimaryRoot denote four-year-old fine-root and primary-root samples. The heatmap shows $\log_2$(TPM + 1) values and retains the fixed Clade I–V gene order. The side strip indicates subgenome A or B.
